# Supplementary material for: Real-time monitoring of superoxide anion radical generation in response to wounding: electrochemical study
Source: PeerJ. 2017 Jul 13;5:e3050. doi: 10.7717/peerj.3050 (PMC5527980; doi:10.7717/peerj.3050)
Supplement: Data S1 — I. Photographs of glass capillary (4 nos.) each with an inner diameter of about 1.2mm and wall thickness of 200µm used for inducing mechanical injury in spinach leaves. II. (A) Photograph of Spinach leaves in the experimental setup. For each measurement, young spinach leaves of size approximately 8-10mm were chosen. (B) Photographs of petriplate with Spinach leaves showing the arrangement of electrodes. III. Photographs of Spinach leaves showing its state after mechanical injury with glass capillary. Injury was made 1 time (A), 5 times (B) and 20 times (C). IV. Kinetics of the production of O2•− measured using polymeric iron porphyrin based modified carbon electrode during wounding in spinach leaves (arrow indicates mechanical wounding). The wounding in spinach leaves was done 20 times close to the site of electrode during the measurement and oxidation current for O2•− was measured. [file peerj-05-3050-s001.pdf]

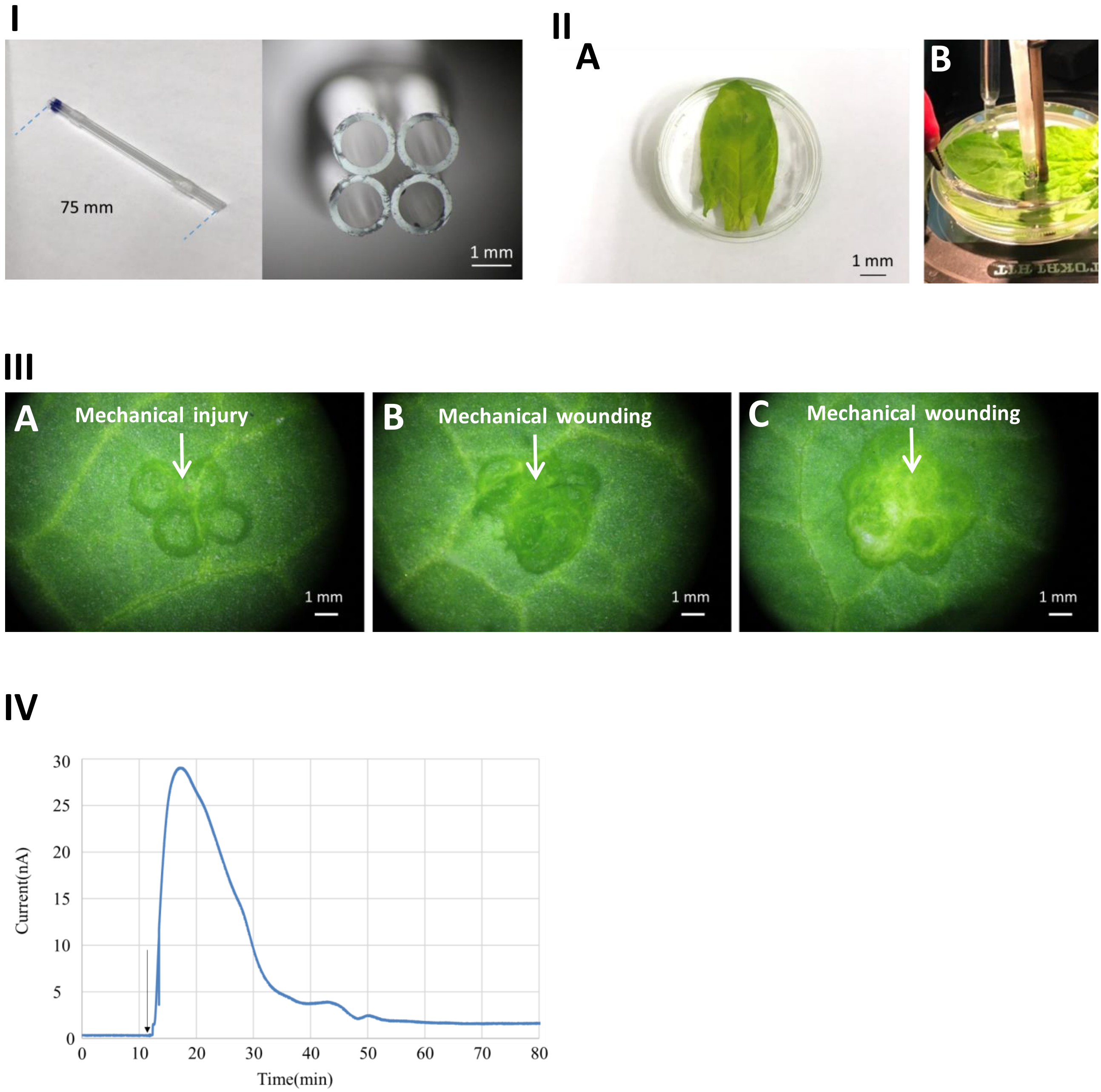

Supplementary data 1: I. Photographs of glass capillary (4 nos.) each with an inner diameter of about 1.2mm and wall thickness of 200µm used for inducing mechanical injury in spinach leaves. **II.** (A) Photograph of Spinach leaves in the experimental setup. For each measurement, young spinach leaves of size approximately 8-10mm were chosen. (B) Photographs of petriplate with Spinach leaves showing the arrangement of electrodes. **III.** Photographs of Spinach leaves showing its state after mechanical injury/wounding with glass capillary. Injury was made 1 time (A), 5 times (B) and 20 times (C). **IV.** Kinetics of the production of $O_2^{\bullet -}$ measured using polymeric iron porphyrin based modified carbon electrode during wounding in spinach leaves (arrow indicates mechanical wounding). The wounding in spinach leaves was done 20 times close to the site of electrode during the measurement and oxidation current for $O_2^{\bullet -}$ was measured.
