# Supplementary figures and images for: Real-time monitoring of superoxide anion radical generation in response to wounding: electrochemical study
Source: PeerJ. 2017 Jul 13;5:e3050. doi: 10.7717/peerj.3050 (PMC5527980; doi:10.7717/peerj.3050)

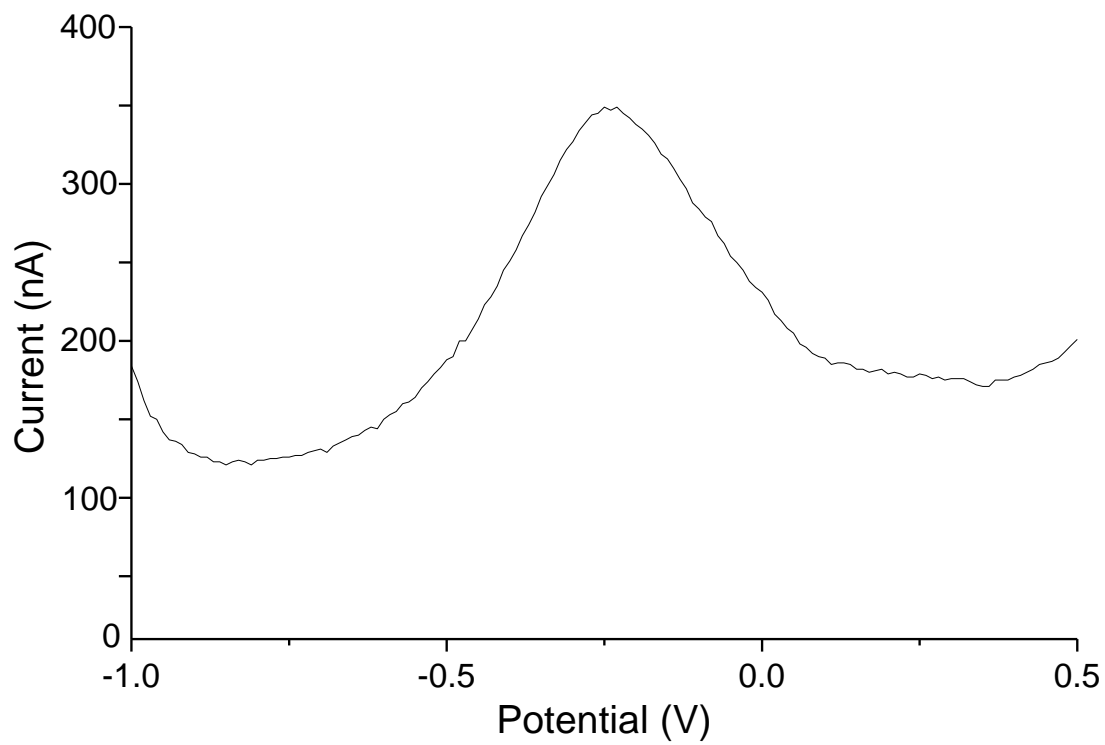

Supplement: Data S2 — DPV for the electropolymerized [Fe(im)2(ttp)]Br complex recorded in an aqueous electrolyte solution containing phosphate buffer (pH 7.2). [file peerj-05-3050-s002.pdf]

**A**

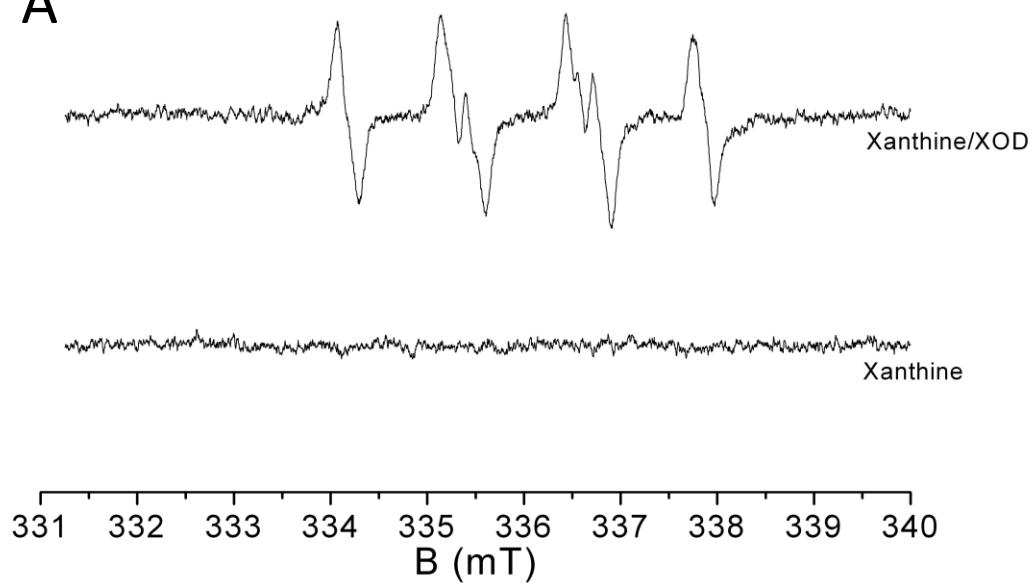

**B**

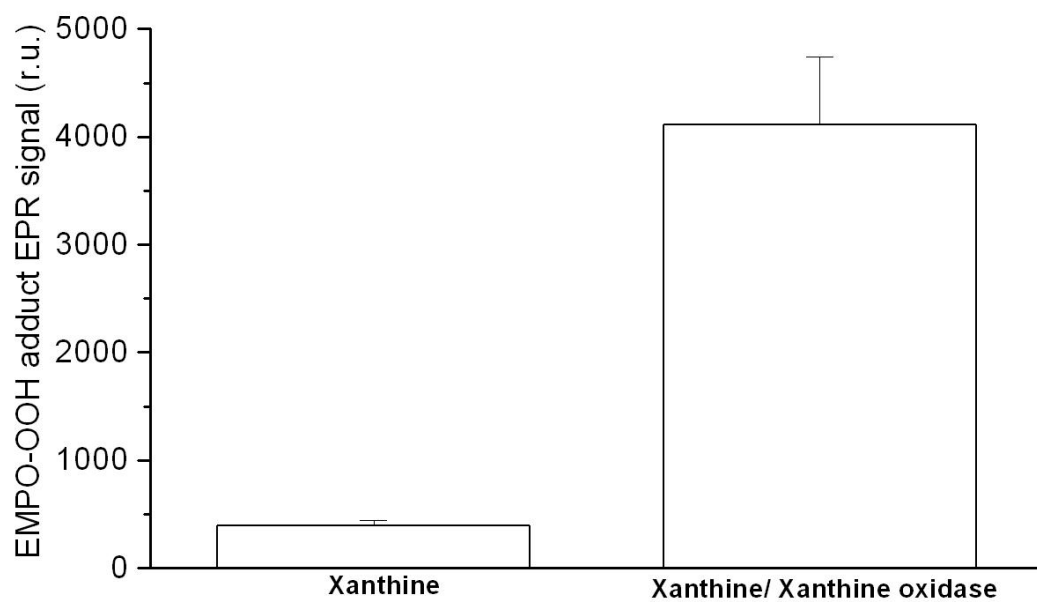

Supplement: Data S3 — EMPO-OOH adduct EPR spectra were measured in the presence of 25 mM EMPO, 40 mM phosphate buffer (pH 7.2) in the absence and presence of xanthine (1 mM) and xanthine oxidase (0.2 U ml−1). Panel (A) shows the spectra measured in the phosphate buffer only (trace a) and X/XO system (trace b); (B) mean value ±SD, n = 3, of the EMPO-OOH adduct EPR spectra. The intensity of the EPR signal depicted in (B) was evaluated by measuring the relative height of the central peak of the first derivative of the EPR absorption spectrum. [file peerj-05-3050-s003.pdf]

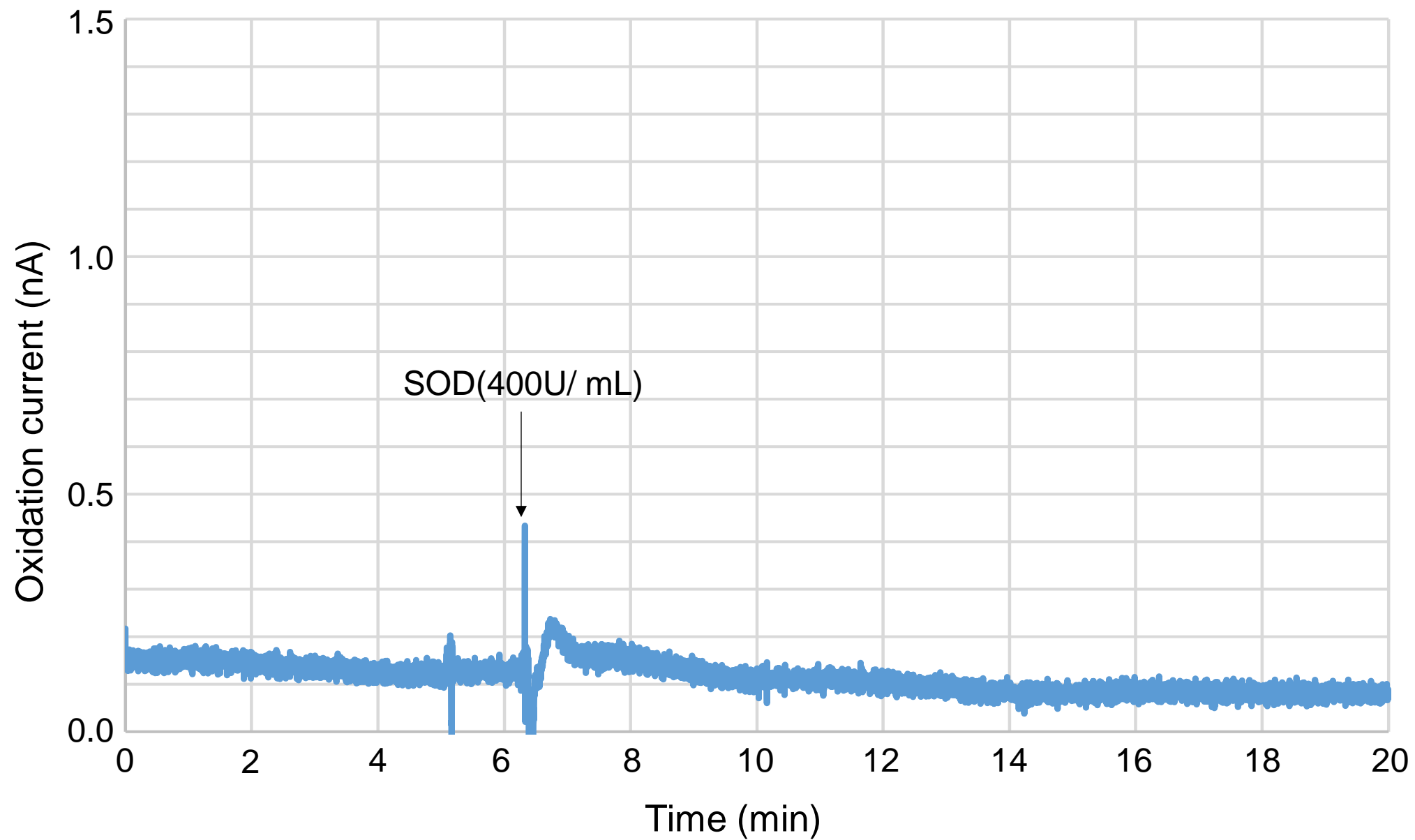

Supplement: Data S4 [file peerj-05-3050-s004.pdf]
